# Comparative Evaluation of Surface Hardness of Bis-Acryl Composite and Polymethyl Methacrylate-Based Provisional Restorative Materials: An In Vitro Study

**DOI:** 10.7759/cureus.83269

**Published:** 2025-04-30

**Authors:** Kartik Sokhal, Sandeep Kumar, Rajnish Aggarwal, Iqbal Kaur, Bhavna Thoidingjam, Aniruddha Deokate, Kulashekar R Nandalur

**Affiliations:** 1 Department of Prosthodontics, Surendera Dental College and Research Institute, Sri Ganganagar, IND; 2 Department of Prosthetic Dental Sciences, College of Dentistry, Jazan University, Jazan, SAU

**Keywords:** acrylic resins, hardness, polymethyl methacrylate, provisional, restorations

## Abstract

Introduction: Provisional restorations are essential components of fixed prosthodontic treatment that provide protection, function, esthetics, and guidance during the transitional phase before definitive restorations are placed. The choice of materials for provisional restorations plays a critical role in determining their clinical effectiveness and durability. Among the numerous available materials, bis-acryl composite resins and polymethyl methacrylate (PMMA)-based acrylics are widely used. Surface hardness is a key mechanical property that directly affects a material's resistance to masticatory forces, wear, and deformation during function. This in vitro study was conducted to compare the surface hardness of three bis-acryl composite materials with that of one PMMA-based material to aid clinicians in selecting the most suitable material based on the performance characteristics.

Materials and methods: This in vitro study was conducted in the Department of Prosthodontics. Group 1 (n=40) used 3M Protemp 4 Temporization Material (St. Paul, MN: 3M ESPE AG), group 2 (n=40) used Dentsply Integrity Temporary Crown and Bridge Material (York, PA: Dentsply Sirona), group 3 (n=40) used Vericom Care C&B Dual-Cure Temporary Material (Anyang, South Korea: Vericom Co. Ltd.), and group 4 (n=40) used Pyrax High-Quality Cold Cure SC-10 (Roorkee, India: Pyrax Polymars). The specimens were prepared using stainless-steel molds. Each material was manipulated according to the manufacturer's instructions and cured according to recommended protocols. Vickers hardness testing was performed under a 25 g load for 15 s. Two indentations were made per specimen, and the Vickers Hardness Number (VHN) was calculated. The data were then subjected to statistical analysis.

Results: Group 1 recorded the highest mean hardness (20.54±0.69 VHN), followed by group 2 with 19.80±0.47 VHN, group 3 with 18.55±1.36 VHN, and group 4 showing the lowest hardness (16.65±0.66 VHN). One-way Analysis of Variance (ANOVA) revealed statistically significant differences between all groups (p<0.01), with group 1 exhibiting the highest hardness across pairwise comparisons.

Conclusion: Bis-acryl composite materials demonstrated a significantly higher surface hardness than PMMA-based materials. 3M Protemp 4 showed the greatest hardness and may be best suited for long-term provisional restorations, whereas Pyrax SC-10 is more appropriate for short-term use owing to its lower mechanical strength.

## Introduction

Provisional restorations play a critical role in restorative and prosthetic dentistry by temporarily replacing missing or prepared tooth structures until the final restoration is achieved. These restorations serve multiple purposes, including protecting prepared teeth, maintaining occlusal stability, safeguarding exposed dentin, and preserving overall function and patient comfort during the interim period [1]. In addition to their protective function, they contribute to esthetics and phonetics, allowing patients to maintain normal speech and appearance while awaiting definitive restorations. Given their temporary nature, provisional materials must balance ease of manipulation with adequate mechanical properties to withstand the functional demands of the oral environment [2].

One of the most significant mechanical properties that determines the clinical performance of provisional restorative materials is their surface hardness [3]. Hardness reflects the ability of a material to resist surface deformation, wear, and abrasion. In the oral cavity, where restorations are exposed to continuous mechanical loading from mastication and parafunctional habits, the hardness of a restoration is a key factor in its durability [4]. Materials that lack sufficient hardness may experience rapid wear or surface breakdown, leading to occlusal discrepancies, compromised marginal adaptation, and potential patient discomfort. However, materials with higher hardness values tend to resist wear more effectively and maintain their shape and integrity over time, making them more suitable for long-term provisional applications [5].

The Vickers hardness test is one of the most widely used methods for evaluating the surface hardness of dental materials including provisional restorations [6]. It provides a standardized and reliable means of measuring a material’s resistance to indentation, thereby offering insights into its ability to withstand masticatory forces. Several factors influence the hardness of provisional materials, such as the chemical composition, polymerization method, and exposure to external conditions such as temperature and moisture. Therefore, it is essential to conduct comparative evaluations of different commercially available materials under controlled conditions to determine the materials that offer the most favorable performance characteristics [7].

Provisional restorative materials are generally categorized into the following two main types: methacrylate-based resins such as polymethyl methacrylate (PMMA) and bis-acryl composites. Methacrylate-based resins are traditionally favored because of their affordability, ease of handling, and long history of clinical use [3]. However, they tend to exhibit a lower surface hardness and greater susceptibility to wear and degradation over time [3]. In contrast, bis-acryl composites have emerged as popular alternatives because of their superior mechanical properties, including enhanced hardness and flexural strength. Despite their advantages, bis-acryl materials are brittle and prone to fracture if not handled carefully [8].

Previous studies have shown that bis-acryl materials generally exhibit higher hardness values than methacrylate-based resins both immediately after fabrication and after periods of aging [3,8,9]. However, hardness can also be affected by the method of polymerization; light-cured materials often demonstrate higher hardness than self-cured materials, and by storage conditions. Immersion in water or artificial saliva can lead to a gradual decrease in mechanical properties due to plasticization and material degradation. Despite these findings, there remains a need for comprehensive comparative studies that evaluate the hardness of multiple provisional materials under standardized laboratory conditions, as most of the available research focuses on isolated products.

This in vitro study aimed to address this gap by comparing the Vickers hardness of various commercially available provisional restorative materials, including bis-acryl and methacrylate-based options. The objective of this study was to determine which material exhibited the highest hardness and, by extension, might offer the most durable clinical performance in provisional restorations.

## Materials and methods

The present in vitro study was conducted in the Department of Prosthodontics, Surendera Dental College and Research Institute, Sri Ganganagar, India. The study was designed as a comparative experimental investigation under standardized laboratory conditions to minimize variables and ensure reproducibility. As this was an in vitro study involving no human participants, tissues, or animals, no ethical clearance was required.

To ensure adequate power and statistical reliability, the sample size was calculated using G*Power version 3.1.9.7 (Düsseldorf, Germany: Heinrich-Heine-Universität), applying an Analysis of Variance (ANOVA, fixed effects, one-way) test with 95% confidence interval and 95% power. The parameters used included an effect size (f) of 0.40, a significance level (α) of 0.05, and four comparison groups. The calculated total sample size was 112; however, to increase robustness and account for variability or potential data loss, 160 specimens were prepared (40 per group).

A stainless-steel die was fabricated in accordance with American Dental Association (ADA) specification no. 27, measuring 30 mm in length, 10 mm in width, and 2 mm in thickness. The mold was created using a three-pour technique with a type III dental stone (Tokyo, Japan: GC Corp.) and a standard denture-processing metal flask to ensure consistent mold formation and avoid air bubble entrapment. A thin layer of the separating medium was applied to the die before flasking. Once the mold was finalized and deflasked, the metal pattern was removed, and a mold cavity was used to fabricate the test specimens.

The four provisional restorative materials evaluated included group 1 (n=40) 3M Protemp 4 Temporization Material (St. Paul, MN: 3M ESPE AG), a bis-acryl resin-based material containing bifunctional methacrylates; group 2 (n=40) Dentsply Integrity Temporary Crown and Bridge Material (York, PA: Dentsply Sirona), a bis-acryl resin-based material with multifunctional methacrylates; group 3 (n=40) Vericom Care C&B Dual-Cure Temporary Material (Anyang, South Korea: Vericom Co. Ltd.), a dual-cured bis-acryl resin-based material containing methacrylates; and group 4 (n=40) Pyrax High Quality Cold Cure SC-10 Tooth-Colored Self-Cure Temporary Acrylic Crown and Bridge Material (Roorkee, India: Pyrax Polymars), and a PMMA-based resin.

Each material was prepared according to the manufacturer’s instructions. In groups 1 and 2, the bis-acryl materials were dispensed into the molds using a Garant Dispenser (St. Paul, MN: 3M ESPE AG) at a 10:1 ratio and subjected to a hydraulic press at 30 psi for five minutes. Group 3 material was dispensed using a DS50 Extruder Gun (St. Paul, MN: 3M ESPE AG) at a 1:1 ratio, light-cured for 40 s, and post-cured for 10 min. The PMMA-based material in group 4 was manually mixed and allowed to self-cure at room temperature for 15 min. After curing, all the specimens were trimmed and finished using tungsten carbide burs or abrasive discs to ensure uniform surface conditions.

To ensure reliability, all procedures were performed by a single calibrated operator trained in specimen preparation and testing. Prior to the main study, a pilot phase was conducted to confirm the consistency of the specimen fabrication and hardness testing. A Vickers Hardness Testing Machine (HV-1000B; Yantai, China: Shancai Testing Instruments Co. Ltd.) was calibrated before each testing session using a standard reference block to maintain the measurement accuracy. Each specimen underwent two separate indentations using a Vickers diamond indenter under a load of 25 g for 15 s. The indentations were spaced at least 2 mm apart to avoid overlapping or interference. The diagonal lengths of the indentations were measured, and the Vickers Hardness Number (VHN) was calculated using the formula VHN = 1.8544 × F/d², where F represents the applied force (0.025 kgf) and d is the mean diagonal length of the indentation.

The collected data were statistically analyzed using SPSS software version 25.0 (Armonk, NY: IBM Corp.). Descriptive statistics were used to summarize the means and standard deviations of the VHN values for each group. One-way ANOVA was employed to determine significant differences in hardness between the groups, and post-hoc Tukey's Honestly Significant Difference (HSD) test was applied for pairwise comparisons to evaluate the mean difference (MD) at a 95% confidence interval (CI). The significance level was set at p<0.05.

## Results

The highest mean hardness was noted in group 1 (20.54±0.69 VHN), followed by group 2 (19.80±0.47 VHN) and group 3 (18.55±1.36 VHN). Group 4 (16.65±0.66) showed the lowest mean hardness (Table 1). One-way ANOVA revealed statistically significant differences (p<0.01) between the groups (Table 2).

**Table 1:** Descriptive statistics for hardness of four groups in Vickers Hardness Number (VHN). SE: standard error; CI: confidence interval Data are presented in the form of mean±SD.

Groups	Mean±SD	SE	Mean at 95% CI		Minimum	Maximum
			Lower bound	Upper bound		
3M Protemp 4	20.54±0.69	0.11	20.32	20.76	19.60	21.81
Dentsply Integrity	19.80±0.47	0.07	19.65	19.95	18.77	20.29
Vericom Care C&B	18.55±1.36	0.21	18.12	18.99	15.85	21.79
Pyrax SC-10	16.65±0.66	0.10	16.44	16.86	15.01	17.89

**Table 2:** Intergroup comparison of mean hardness values Vickers Hardness Number (VHN) among the groups using one-way Analysis of Variance (ANOVA). *P-value <0.05 was considered significant.

Groups	Sum of squares	Difference	Mean square	Maximum	p-Value
Intergroup comparison	347.58	3	115.86	21.81	<0.01*

The data indicated that group 4 had the lowest hardness values. Conversely, group 1 exhibited the highest hardness, showing a positive mean difference compared with all other materials. Among the pairwise comparisons, the largest mean difference was between groups 1 and 4 (MD: 3.892, CI: 3.3906-4.3925), indicating a significant difference in hardness. The smallest significant difference was observed between groups 1 and 2 (MD: 0.740, CI: 0.2394-1.2413). As all p-values were <0.01, the differences between the groups were statistically significant. The results confirmed that 3M Protemp 4 had the highest hardness, followed by Dentsply Integrity, Vericom Care C&B, and Pyrax SC-10 with the lowest hardness. These findings suggest that material selection is crucial in applications requiring high hardness values, as Pyrax SC-10 may be less durable than the other materials (Table 3).

**Table 3:** Pairwise comparison of mean hardness values among the groups. *P-value <0.05 was considered significant. The “mean difference” was calculated by subtracting the mean hardness value of the first group from the mean hardness value of the second group in each pairwise comparison. A negative value indicates that the second group has a higher mean hardness, while a positive value indicates that the first group has a higher mean hardness.

Pairwise groups		Mean difference	Standard error	p-Value using post-hoc Tukey's test	95% Confidence interval	
					Lower bound	Upper bound
3M Protemp 4	Dentsply Integrity	0.74	0.19	0.001*	0.24	1.24
	Vericom Care C&B	1.99	0.19	<0.01	1.49	2.49
	Pyrax SC-10	3.89	0.19	<0.01	3.39	4.39
Dentsply Integrity	3M Protemp 4	-0.74	0.19	0.001*	-1.24	-0.24
	Vericom Care C&B	1.25	0.19	<0.01	0.75	1.75
	Pyrax SC-10	3.15	0.19	<0.01	2.65	3.65
Vericom Care C&B	3M Protemp 4	-1.99	0.19	<0.01	-2.49	-1.49
	Dentsply Integrity	-1.25	0.19	<0.01	-1.75	-0.75
	Pyrax SC-10	1.90	0.19	<0.01	1.40	2.40
Pyrax SC-10	3M Protemp 4	-3.90	0.19	<0.01	-4.39	-3.39
	Dentsply Integrity	-3.15	0.19	<0.01	-3.65	-2.65
	Vericom Care C&B	-1.90	0.20	<0.01	-2.40	-1.40

## Discussion

Provisional restorations constitute a fundamental aspect of fixed prosthodontic therapy, acting as a temporary yet essential measure throughout the interval between tooth preparation and installation of the definitive prosthesis. The choice of a suitable material for the creation of provisional restorations involves a complex decision-making process that requires thorough evaluation of various physical, mechanical, and handling characteristics. Critical considerations include the flexural strength, surface hardness, resistance to wear, dimensional stability, polymerization shrinkage, wettability, and color stability. These characteristics guarantee that provisional restoration is capable of enduring functional loads, resisting wear, and preserving its structural integrity during the transitional phase [3].

Hardness is a critical characteristic of provisional restorative materials as it directly affects their capacity to endure masticatory forces, resist abrasive wear, and preserve structural integrity throughout the interim period. For example, Diaz-Arnold et al. underscored that provisional materials must demonstrate sufficient hardness to guarantee clinical efficacy, particularly in areas subjected to high stress, such as the posterior dentition [10]. Similarly, Gratton and Aquilino asserted that hardness is a pivotal determinant in assessing the durability and functionality of provisional restorations [11].

The findings of our study indicate that 3M Protemp 4 exhibited the highest hardness value, followed closely by Dentsply Integrity, whereas Vericom Care C&B and Pyrax SC-10 had comparatively lower hardness values. The trend observed in this study is consistent with the findings of Jo et al. and Savabi et al., who reported that bis-acryl composites generally exhibit higher hardness than traditional PMMA resins [12,13]. This supports the superior performance of 3M Protemp 4 and Dentsply Integrity, both of which are bisacryl-based materials. Their enhanced hardness can be attributed to their cross-linked polymer matrix and the incorporation of inorganic fillers, which improve the resistance to wear and surface degradation. Conversely, the lower hardness of Pyrax SC-10, a PMMA-based material, aligns with the studies conducted by Thompson and Luo, which highlighted that PMMA materials tend to have lower hardness owing to their chemical composition and polymerization process [14]. These materials are generally easier to manipulate and more cost-effective but may not provide the same level of wear resistance as bis-acryl composites. This suggests that while PMMA-based materials remain viable for short-term use, bis-acryl composites may be more suitable for extended provisional applications that require enhanced mechanical strength.

Kerby et al. and Knobloch et al. found that bis-acryl resins, which contain multifunctional methacrylates, exhibit higher hardness and fracture toughness compared to urethane-based materials [15,16]. This is consistent with the performance of the Vericom Care C&B DC Temporary Material in this study, which demonstrated superior hardness due to its bis-acryl resin composition. In contrast, Pyrax High Quality Cold Cure SC-10, a PMMA-based material, exhibited lower hardness, which is consistent with the findings of Thompson and Luo, who reported that PMMA materials are generally less hard but easier to manipulate [14]. The hardness of provisional materials can also be influenced by the fabrication techniques and storage conditions. Rodríguez-Guardado et al. and Takamizawa et al. emphasized that proper polymerization and post-polymerization conditioning are critical for achieving optimal hardness [8,17]. For instance, the mixing process of materials, such as Pyrax SC-10, plays a significant role in determining the final hardness and overall performance of the restoration. Pyrax SC-10, a PMMA-based material, is typically supplied as a powder-liquid system that requires careful proportioning and thorough mixing to achieve homogeneous consistency. Inadequate mixing can lead to incomplete polymerization, resulting in reduced hardness and compromised mechanical properties.

Water immersion can reduce the hardness of provisional materials over time, highlighting the importance of proper storage to maintain their mechanical properties. This is particularly relevant for materials such as Pyrax SC-10, which may be sensitive to moisture during the initial setting phase [18]. Proper handling, including avoiding premature exposure to water or saliva, is essential for ensuring optimal hardness and longevity. Additionally, post-polymerization conditioning, such as storing the material in a dry environment or using light-curing units for dual-cure materials, can further enhance the hardness and stability.

The preparation of Pyrax SC-10, a PMMA-based provisional restorative material, involves combining the powder and liquid components in a 2:1 ratio by volume to achieve a dough-like consistency that is suitable for molding and adaptation. The accuracy of this mixing process is crucial, as overmixing can introduce air bubbles, whereas undermixing may result in incomplete polymerization, both of which can negatively impact the hardness and overall mechanical integrity of the material. Clinicians must carefully follow the manufacturer’s guidelines to ensure optimal results and consistency in material performance. Additionally, the working time of Pyrax SC-10 was approximately 2-3 min, allowing adequate manipulation before the setting reaction was initiated. However, efficient handling is essential to avoid premature setting, which can compromise the polymerization uniformity and lead to surface inconsistencies. Given its lower hardness values, Pyrax SC-10 may be more suitable for short-term provisional applications, where ease of modification and cost-effectiveness are prioritized over long-term durability [19].

The findings of this study have several significant clinical implications. Materials with higher hardness values, such as 3M Protemp 4 and Dentsply Integrity, are more suitable for long-term provisional restorations, particularly in patients with high occlusal force or parafunctional habits. However, as noted by Young et al., harder materials may be more challenging to adjust and polish, which could affect their clinical utility [20]. Therefore, clinicians must balance hardness with other properties such as ease of manipulation and esthetics when selecting provisional materials. The results of this study are consistent with those of Pasha et al. and Agroya et al., who compared the hardness of various provisional materials and found that bis-acryl resins generally exhibit superior mechanical properties [21,22]. Astudillo-Rubio et al. conducted a systematic review and meta-analysis and concluded that hardness is a critical factor in determining the clinical performance of provisional materials [3]. The findings of this study further validate these conclusions and provide additional insight into the performance of commercially available materials.

Clinical significance of the study

Provisional restorations play a crucial role in fixed prosthodontics by maintaining protection, function, and guiding the final prosthesis. Material selection, particularly hardness, significantly influences the durability and clinical performance. Bis-acryl composites such as 3M Protemp 4, Dentsply Integrity, and Vericom Care C&B exhibit superior hardness, making them well suited for long-term use in high-stress areas. Variations in hardness among these materials are likely due to differences in the filler content, resin composition, and polymerization efficiency. Conversely, PMMA-based materials such as Pyrax SC-10 are more affordable and easier to modify, making them ideal for short-term applications; however, their lower hardness requires cautious use. Proper polymerization is essential for reducing residual monomers and improving mechanical strength. Although bis-acryl materials offer excellent strength, their limited adjustability is a drawback. Emerging digital technologies such as computer-aided design/computer-aided manufacturing (CAD/CAM) may present improved alternatives, suggesting the need for further research [23,24]. Material choice should balance strength, adaptability, and clinical requirements.

Limitations

Despite the controlled and standardized methodology employed in this in vitro study, certain limitations must be acknowledged. Primarily, the study was conducted under laboratory conditions that do not fully replicate the complex oral environment, including variations in temperature, pH, saliva, and masticatory forces, which can significantly influence the long-term behavior and wear characteristics of provisional materials. The absence of thermocycling or mechanical loading protocols may limit the extrapolation of the results to clinical scenarios in which dynamic functional stresses are present. Furthermore, only one mechanical property, that is, the surface hardness, was evaluated. Hardness alone does not comprehensively determine the clinical performance of a material. Other properties such as flexural strength, fracture toughness, marginal integrity, and color stability also play crucial roles in determining the suitability of provisional restorations. The use of a single operator, although beneficial for minimizing variability, introduces the potential for operator bias or unintentional inconsistencies. In addition, only four commercially available materials were tested, which may not represent the entire spectrum of provisional materials currently in clinical use. The influence of different fabrication techniques, storage conditions, and aging protocols has not been explored. Future studies incorporating more clinical variables, long-term aging, and patient-centered outcomes are necessary to better understand the comprehensive performance of provisional restorative materials. This study was limited to surface hardness; future research should assess flexural strength, wear resistance, fracture toughness, color stability, and biocompatibility to fully characterize clinical performance.

## Conclusions

It can be concluded that bis-acryl resin-based provisional restorative materials demonstrate significantly higher surface hardness than PMMA-based materials. Among the tested groups, 3M Protemp 4 exhibited the highest mean hardness, followed by Dentsply Integrity and Vericom Care C&B, whereas Pyrax SC-10, a PMMA-based material, showed the lowest hardness. The superior hardness of bis-acryl materials can be attributed to their cross-linked polymer matrices and higher filler content, which makes them more suitable for long-term provisional restorations, especially in high-stress areas. Conversely, PMMA-based materials, although easier to handle and more economical, may be better suited for short-term applications because of their lower wear resistance. These findings emphasize the importance of careful material selection based on clinical requirements, functional demands, and duration of use to optimize the performance and longevity of provisional restorations.
